# Cell-cycle length of medial ganglionic eminence progenitors contributes to interneuron fate

**DOI:** 10.1007/s13238-021-00851-w

**Published:** 2021-05-27

**Authors:** Ni Zong, Min Wang, Yinghui Fu, Dan Shen, Yong-Chun Yu

**Affiliations:** Jing’an District Central Hospital of Shanghai, State Key Laboratory of Medical Neurobiology and MOE Frontiers Center for Brain Science, Institutes of Brain Science, Fudan University, Shanghai, 200032 China

**Dear Editor,**

GABAergic interneurons contribute to cortical function by regulating the balance of activity. Malfunction of inhibitory interneurons has been associated with neuropsychiatric disorders (Hashimoto et al. 2003; Marín 2012). The most striking feature of GABAergic interneurons is their diversity of cell types. Although fate-mapping studies have demonstrated the presence of spatially and temporally distinct progenitor domains for interneuron subclasses within the embryonic ventral telencephalon (Butt et al. 2005; Inan et al. 2012), the mechanisms of interneuron subtype-determination during development remain poorly understood.

Considerable evidence supports the idea that cell fate determination throughout neurogenesis is intimately linked with cell-cycle length (Ohnuma and Harris 2003; Pilaz et al. 2016). In fact, disruption of normal cell-cycle length is known to alter neurogenesis (Hardwick et al. 2015; Boyd et al. 2015). However, due to technical limitations, we still lack detailed information about the specific impact of cell-cycle length on cell fate determination *in vivo*. An important unanswered question is whether there is a direct relationship between cell-cycle length of neural progenitors and interneuron fate determination.

In this study, we developed a triple thymidine analog labeling method to label progenitors undergoing short cycles (PSC) or progenitors undergoing long cycles (PLC) in the medial ganglionic eminence (MGE) *in vivo* (Fig. 1A–C). In brief, we carried out an initial injection of IdU followed 2 h later by an injection of BrdU at embryonic day 13.5 (E13.5). Cells labeled by IdU, but not BrdU (IdU^+^BrdU^−^) had left S phase. After various intervals (4, 9, 12 and 14 h, defined as ΔT), we injected EdU to label cells that re-entered S phase (IdU^+^BrdU^−^EdU^+^) (Fig. 1A). Using this labeling method, the cell-cycle lengths (T_C_) of IdU^+^BrdU^−^EdU^+^ cells can be roughly calculated for different ΔTs (Fig. 1B). We found that the percentage of IdU^+^BrdU^−^EdU^+^ cells in IdU^+^BrdU^−^ cells in ΔT = 9 h group was around three times higher than in the other ΔT groups at E13.5 (Figs. 1D and S1A–H; Table S1); whereas, the peak of ΔT at E15.5 was 12 h (Figs. 1E and S1I–P; Table S1), longer than at E13.5, suggesting that the average cell-cycle length of MGE progenitors gradually increases during embryonic development. To further characterize the variability of cell-cycle length, we used time-lapse microscopy to monitor cell-cycle progression in cultured MGE cells at E13.5 (Fig. S2A–C). Histogram analysis showed that the distribution of cell-cycle lengths of MGE progenitors occupied a wide range, between 8 and 22 h (Fig. S2D). The average cell-cycle length of cultured MGE cells at E13.5 was 12.20 ± 0.20 h, which was significantly shorter than at E15.5 (20.11 ± 1.16 h) (Fig. S2E; Table S1). In addition, we found that only 24.0% of lineages showed relatively stable cell-cycle length in 3-round divisions (relative variation less than 10%) (Fig. S2F and S2G). These results suggest that cell-cycle length in individual lineages of MGE progenitors over multiple divisions exhibits remarkable heterogeneity and instability.

**Figure 1:**
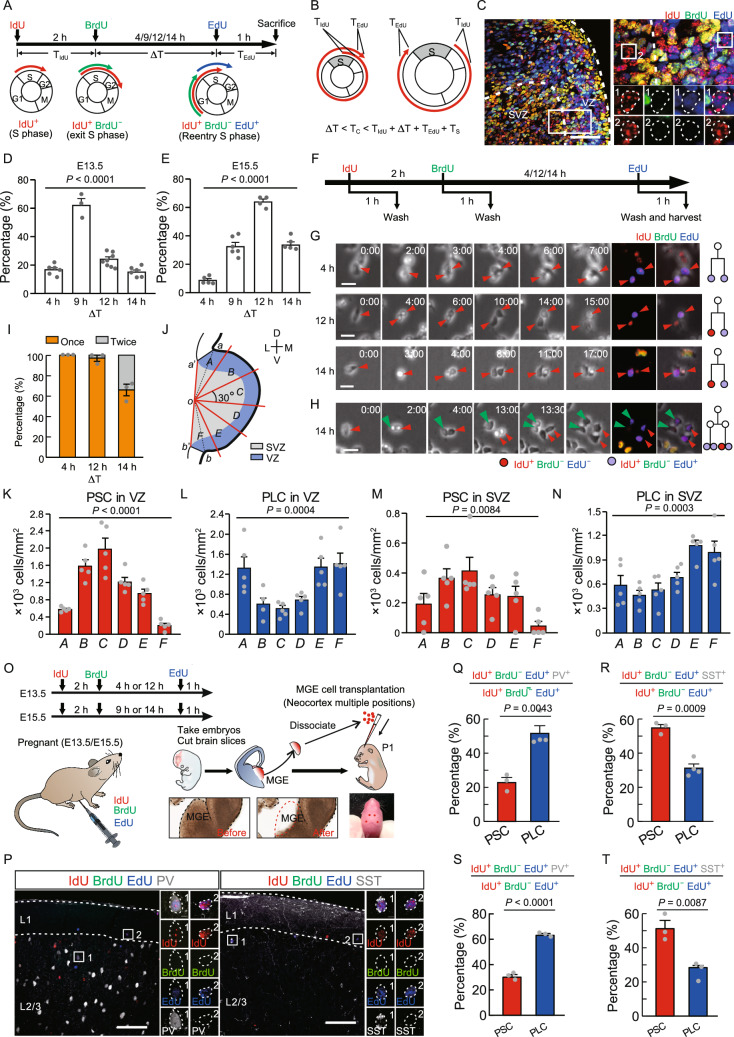
**MGE progenitor cells with different cell-cycle lengths are biased toward generating specific cortical interneuron subgroups**. (A) Experimental outline. Pregnant mice at E13.5 and E15.5 received an injection of IdU and 2 h later BrdU, followed by an injection of EdU at various intervals (4, 9, 12 or 14 h; ΔT). Brains were collected for analysis 1 h after EdU injection. (B) The estimated range of cell-cycle lengths for a specific ΔT. (C) Representative image of thymidine analog-treated MGE at E13.5 (ΔT = 4 h), magnified images on the right (rectangular region). Cell 1 is IdU^+^BrdU^−^EdU^+^, and cell 2 is IdU^+^BrdU^−^EdU^−^. Scale bar, 50 μm. (D and E) The percentage of IdU^+^BrdU^−^EdU^+^ cells in IdU^+^BrdU^−^ cells in E13.5 and E15.5 MGE under different ΔT conditions. (F) Experimental outline in cultured MGE cells. (G and H) Representative time-lapse and immunostained images of cultured MGE cells for different ΔT conditions (4 h, 12 h, 14 h). Scale bar, 20 μm. (I) Bar plot showing frequency of division for IdU^+^BrdU^−^EdU^+^ cells during recording under different ΔT conditions. Data are presented as mean ± SEM. (J) Schema depicting the partitioning of the MGE into six sectors (See Supplementary Materials). (K–N) The density of PSC and PLC in A–F VZ sectors and A–F SVZ sectors. (O) Experimental outline showing thymidine analog injections and MGE cell transplantation and the anatomical structures from which the embryonic tissues were harvested for transplantation. (P) Representative images showing thymidine analogs co-labeling with PV (left panel, ΔT = 4 h) or SST (right panel, ΔT = 4 h) in the cortex of P30 host mice. Cell 1 and 2 in the left panel are IdU^+^BrdU^−^EdU^+^PV^+^ and IdU^+^BrdU^−^EdU^+^PV^−^, respectively. Cell 1 and 2 in the right panel are IdU^+^BrdU^−^EdU^+^SST^+^ and IdU^+^BrdU^−^EdU^+^SST^−^, respectively. Scale bar, 100 μm. (Q and R) Quantification of the percentage of PSC and PLC that were positive for PV or SST when transplanted at E13.5. (S and T) Quantification of the percentage of PSC and PLC that were positive for PV or SST when transplanted at E15.5. Data are presented as mean ± SEM

To determine whether IdU^+^BrdU^−^EdU^+^ cells do indeed undergo division, and if so, how many times they divide, we monitored the lineages of cultured MGE cells at E13.5, and sequentially applied IdU, BrdU and EdU into the culture medium to mimic the triple-labeling method *in vivo* (Fig. 1F and 1G). Indeed, for each ΔT, 100% of IdU^+^BrdU^−^EdU^+^ cells divided at least once during observation (Figs. 1G–I and S2H; Table S1). However, in ΔT = 14 h group, 34.05% ± 5.66% of IdU^+^BrdU^−^EdU^+^ cells divided twice (Fig. 1H and 1I; Table S1), suggesting that about one-third of IdU^+^BrdU^−^EdU^+^ cells in ΔT = 14 h group were progenitors undergoing short cycles. During measuring the cell-cycle length of MGE progenitors in the different ∆T groups, we found that the ranges of cell-cycle length for 10%–90% progenitors in the ∆T = 4 h, ∆T = 9 h and ∆T = 12 h groups were 6–12 h, 11–15 h and 13–18 h, respectively (Fig. S2I and S2J; Table S1). Taken together, these results indicate that, for ΔT ≤ 12 h, the triple-labeling method can specifically label MGE progenitors with different cell-cycle lengths at E13.5, and the progenitors labeled in the ∆T = 4 h and ∆T = 12 h have distinct cell-cycle lengths.

As mentioned above, PSC and PLC in MGE at E13.5 can be individually labeled *in vivo* by the triple-labeling method when ΔT = 4 h and ΔT = 12 h. We divided the MGE in coronal brain slices into six 30° sectors (Fig. 1J), the density of IdU^+^BrdU^−^ cells exhibited no significant difference across the six regions under ΔT = 4 and 12 h conditions (Fig. S3A–D; Table S1). Interestingly, the density of PSC in the middle of the ventricular zone (VZ) was significantly higher than in the dorsal and ventral VZ (Fig. 1K; Table S1). In contrast, the density of PLC in the middle of the VZ was dramatically lower than in the dorsal and ventral VZ (Fig. 1L; Table S1). In the subventricular zone (SVZ), we observed a similar bias in PSC and PLC distribution as in the VZ (Fig. 1M and 1N; Table S1). These results suggested that PSC mainly locate in the middle MGE (mMGE), whereas PLC predominantly distribute in the dorsal (dMGE) and ventral MGE (vMGE). We also found that the density of PSC in the caudal MGE was significantly higher than in the rostral and central MGE; whereas the density of PLC in the rostral MGE was significantly higher than in the central and caudal MGE (Fig. S3I–K; Table S1). Several studies have identified two main classes of neural progenitors in the MGE: apical progenitors (APs) which divide along the ventricular surface and basal progenitors (BPs) which divide within the SVZ (Arai et al. 2011). To examine the distribution of PSC and PLC across these two subtypes of neural progenitors, we labeled APs and BPs with Olig2 and cyclin-D2 (CCND2). We found that the percentage of IdU^+^BrdU^−^EdU^+^Olig2^+^ VZ cells in IdU^+^BrdU^−^EdU^+^ VZ cells was comparable between PSC and PLC (Fig. S3L and S3M; Table S1). However, there was a higher percentage of IdU^+^BrdU^−^EdU^+^ CCND2^+^ SVZ cells in the PLC than in the PSC (Fig. S3N and S3O; Table S1), suggesting that there are more PLC than PSC in MGE BPs at E13.5.

In this study, the terms PSC and PLC are based on a single cycle. Several lines of evidence suggest that transplanted MGE cells undergo their final division and exit the cell cycle after transplantation into the host brain (Valcanis and Tan 2003; De la Cruz et al. 2011). To study the relationship between the ‘single round’ cell-cycle length of MGE progenitors and interneuron subgroup fate determination, we transplanted MGE cells from triple thymidine analog-injected mice at E13.5 and E15.5 into the neocortex of postnatal day 1–2 (P1–P2) hosts (Fig. 1O). PSC and PLC at E13.5 were labeled under ΔT = 4 h and ΔT = 12 h conditions; at E15.5, PSC and PLC were labeled under ΔT = 9 h and ΔT = 14 h conditions. In the host cortex, PSC- and PLC-derived interneurons were identified as IdU^+^BrdU^−^EdU^+^. To examine the differentiation of MGE progenitors, we injected thymidine analogs into green fluorescent protein positive (GFP^+^) donor mice. Thymidine analog-labeled cells displayed a characteristic interneuron morphology, indicating that transplanted thymidine analog-labeled cells can develop into mature interneurons in the host neocortex (Fig. S4A–D). Co-labeling of IdU^+^BrdU^−^EdU^+^ with PV or SST markers (labeling two major non-overlapping interneurons subgroups derived from MGE) was then quantified in host cortical sections at P30 (Fig. 1P). We observed that PLC were two times more likely to give rise to PV^+^ interneurons compared to PSC; in marked contrast, PSC produced roughly twice the number of SST^+^ interneurons compared to PLC at E13.5. (Fig. 1Q and 1R; Table S1). Similar results were obtained in transplantations from E15.5 MGE progenitors into P1 neocortex (Fig. 1S and 1T; Table S1). Together, these results suggest that PSC and PLC are biased toward generating SST^+^ and PV^+^ interneuron subgroups, respectively.

We then asked what factors regulate cell-cycle length of MGE progenitors and contribute to cell-cycle heterogeneity. Protein p27^Kip1^ (also called cyclin-dependent kinase inhibitor 1B, CDKN1B) is an inhibitor of cyclin-dependent kinase involved in the regulation of the cell cycle (Polyak et al. 1994), and widely expressed in MGE progenitors. Thus, we speculate that p27 might be a key factor that regulates the cell-cycle length of MGE progenitors. To investigate this, we generated *Nkx2.1-Cre*^+/−^; *p27*^*fl*/*fl*^ mice (*p27* knockout line, *p27*-cKO) and *Nkx2.1-Cre*^−/−^; *p27*^*fl*/*fl*^ littermates (wild-type line, *p27*-WT) to conditionally deleted the *p27*^*Kip1*^ gene in the MGE (Fig. 2A). *p27* expression in MGE was detected by in situ hybridization at E13.5. We found that *p27* was only removed in MGE progenitors of *p27*-cKO mouse line and not in other domains (Fig. S5A). Using dual thymidine analog labeling approaches (BrdU/EdU) in combination with Ki67 labeling at E13.5, we observed that the average T_C_ of MGE progenitors in *p27*-cKO at E13.5 was significantly shorter than in *p27*-WT (Fig. 2B; Table S2). Moreover, we found that the percentage of PSC at E13.5 in *p27*-cKO mice was around two times higher than in *p27*-WT mice (Fig. 2C; Table S2). However, the percentage of PLC in *p27*-cKO mice was significantly lower than in *p27*-WT mice (Fig. 2D; Table S2). These data indicate that deletion of p27 can shorten the cell-cycle length of neural progenitors in the MGE. Accumulated evidence indicates that cleavage plane orientation governs the fate of neural stem cell progeny (Chenn and McConnell 1995; Haydar et al. 2003). To examine whether cell-cycle length regulates mitotic spindle orientation of embryonic MGE progenitors, we calculate the cleavage plane angle of APs (Fig. 2E and 2F). We observed that the fraction of horizontally oriented mitotic spindles (between 0° and 15°) in *p27*-cKO mice were strongly increased as compared with *p27*-WT mice (Fig. 2G). Correspondingly, there was significantly difference in division angles between *p27*-WT and *p27*-cKO mice (Fig. 2H; Table S2). These data indicate that deletion of *p27* can affect mitotic cleavage orientation of neural progenitors in the MGE.

**Figure 2:**
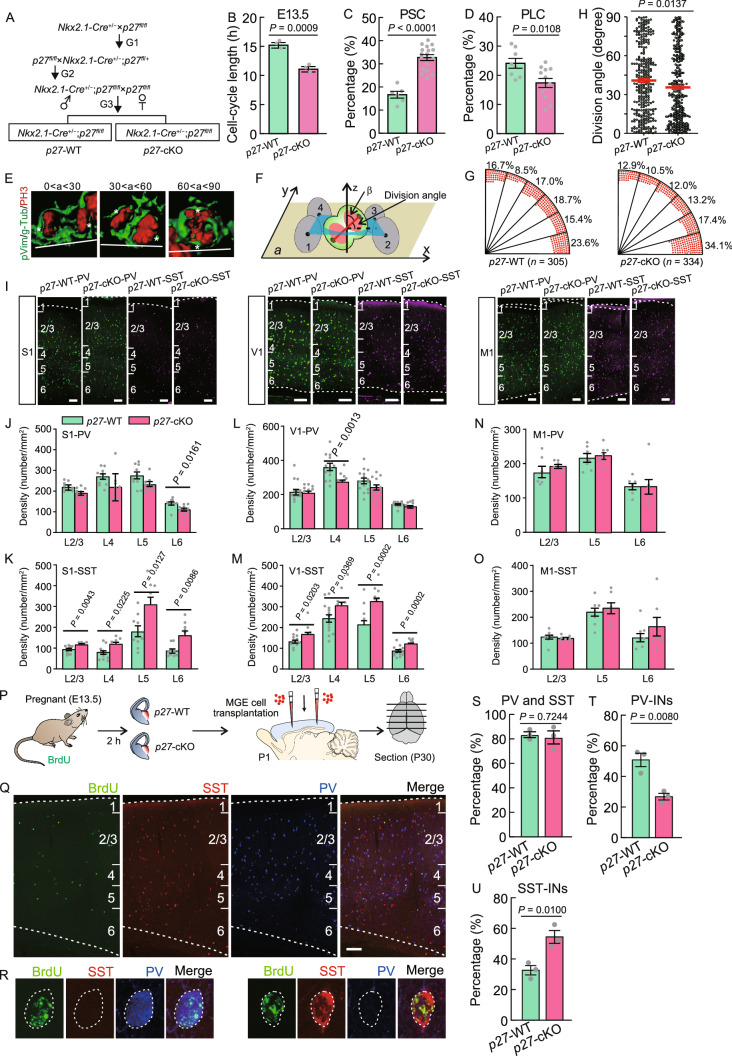
**Deletion of** ***p27*** **shorten the cell-cycle length of MGE progenitors and affect cortical PV**^**+**^ **and SST**^**+**^ **interneuron production.** (A) Illustration of mouse breeding strategy. (B) The average cell-cycle length of E13.5 MGE cells of *p27*-WT or *p27*-cKO mice. (C and D) Quantification of the percentage of PSC and PLC in E13.5 MGE of *p27*-WT mice and *p27*-cKO mice. (E) 3D reconstructions of single-dividing RGCs in the MGE illustrating the three classes of division based on the mitotic cleavage orientation. MGE ventricular surface is at the bottom (white line). Asterisks indicate the centrosomes. (F) Model for the calculation of cleavage plane angle of dividing RGCs in the MGE. Mitotic DNA is in red; the centrosome is in dark green. Cells 1–4 are located at the ventricular surface and adjacent to the targeted cell. Plane *a*, the best-fitting plane for the centers of cells 1–4. β is the angle between the vector connecting the two centrosomes and the normal vector (z axis) of the plane *a*. (G) Distribution of the division angles of MGE progenitor cells in *p27*-WT mice and *p27*-cKO mice. (H) Statistical analysis of division angles between *p27*-WT and *p27*-cKO mice. Data are presented as mean ± SEM. (I) Representative images of PV and SST immunostaining in S1, V1 and M1 from *p27*-WT and *p27*-cKO mice at P30. Scale bar, 100 μm. (J and K) Quantification of the density of PV^+^ and SST^+^ interneurons in S1 of *p27*-WT and *p27*-cKO mice. Each data point represents one brain section. (L and M) Quantification of the density of PV^+^ and SST^+^ interneurons in V1 of *p27*-WT and *p27*-cKO mice. Each data point represents one brain section. (N and O) Quantification of the density of PV^+^ and SST^+^ interneurons in M1 of *p27*-WT and *p27*-cKO mice. Each data point represents one brain section. Data are presented as mean ± SEM. (P) Experimental outline showing transplantation of MGE cells from E13.5 BrdU-injected embryos into the neocortex of P1 neonates. (Q) Representative images showing BrdU co-labeling with PV or SST in the cortex of P30 host mice. Scale bar, 100 μm. (R) Two sample cells showing BrdU co-labeling with PV or SST, respectively. (S) Quantification of the percentage represents the sum of PV^+^BrdU^+^ and SST^+^BrdU^+^ cells in BrdU^+^ cells in *p27*-WT and *p27*-cKO group. (T and U) Quantification of the percentage of transplanted cells from *p27*-WT and *p27*-cKO mice that were positive for PV or SST. Data are presented as mean ± SEM

We then addressed a critical question: is p27 expression necessary for the balance of cortical interneuron subtypes derived from MGE? PV and SST immunoreactivity was analyzed in the primary somatosensory cortex (S1), primary visual cortex (V1), primary motor cortex (M1) and striatum of *p27*-WT and *p27*-cKO mice at P30 (Figs. 2I and S5B–G). We found that the conditional deletion of *p27* in MGE progenitors increased the number of cortical SST^+^ interneurons, but had little effect on PV^+^ interneuron numbers (Figs. 2J–O, S5D and S5G; Table S2). To analyze apoptosis effects of *p27* deletion, we labeled apoptotic cells with cleaved caspase-3 at postnatal day 7 (Fig. S5H), the peak of endogenous interneuron cell death in neocortex (Southwell et al. 2012). We found that *p27*-cKO mice exhibit a similar level of interneuron loss in neocortex as *p27*-WT mice at P7 (Fig. S5I; Table S2). Finally, we queried whether these phenotypes were evident specifically in MGE progenitors at E13.5. We transplanted MGE cells from E13.5 BrdU-injected embryos into the neocortex of P1 neonates (Fig. 2P–R). Interestingly, the percentage of BrdU-expressing transplanted cells that co-labeled with PV or SST did not differ between *p27*-WT and *p27*-cKO groups (Fig. 2S; Table S2), suggesting that deletion of *p27* does not alter MGE progenitors to primarily differentiate into PV^+^ and SST^+^ interneurons. We did not observe PV and SST double positive cells (PV^+^/SST^+^ cells) in transplanted cells. Remarkably, we found that the percentage of PV^+^BrdU^+^ cells in BrdU^+^ cells was reduced by half in the *p27*-cKO group compared to *p27*-WT group (Fig. 2T; Table S2). Conversely, there was about 2-fold higher co-localization of SST with BrdU in the *p27*-cKO group compared to *p27*-WT group (Fig. 2U; Table S2). Of note, compared to transplantation (Fig. 2P–U), there isn’t much of a change in PV^+^ interneuron numbers in the cortex of *p27*-cKO mice at P30 (Fig. 2J and 2L). We speculate that the temporal-spatial change of cell-cycle length of MGE progenitors induced by *p27*-cKO may result in differences in PV^+^ interneuron phenotype in transplantation versus the *p27* mutant. Taken together, these genetic studies demonstrate that p27 as a key factor regulates cortical interneuron fate determination by adjusting cell-cycle length of MGE progenitors.

In summary, we utilized a triple thymidine analog labeling method for the first time to label individual progenitors undergoing short or long cycles *in vivo*. Our data provide direct evidence in support of a causal link between the cell-cycle length of MGE progenitors and cell fate determination of their progeny. We found that PSC mainly give rise to SST^+^ interneurons, whereas PLC generates predominantly PV^+^ interneurons. Moreover, we also identified p27 as a key molecule in regulating cortical interneuron fate determination by adjusting cell-cycle length of MGE progenitors. However, the complete molecular mechanisms are still unclear. Future effort is necessary to understand other molecular and cellular mechanisms by which the cell-cycle length of MGE progenitors influences the fate determination of cortical interneuron subtypes. Elucidating these mechanisms will be crucial for fundamentally understanding interneuron subtype-determination, and for ultimately treating the numerous disorders associated with interneuron dysfunction.

## Supplementary Information

Below is the link to the electronic supplementary material.

Supplementary material 1 (PDF 3197 kb)
