# Supplementary material for: Cell-cycle length of medial ganglionic eminence progenitors contributes to interneuron fate
Source: Protein Cell. 2021 May 27;13(2):141–7. doi: 10.1007/s13238-021-00851-w (PMC8783944; doi:10.1007/s13238-021-00851-w)
Supplement: Supplementary file 1 — Supplementary material 1 (PDF 3197 kb) [file 13238_2021_851_MOESM1_ESM.pdf]

Methods and Materials

Animals

Wild-type CD-1 mice were purchased from Shanghai Laboratory Animal Center (SLAC), CAS, Shanghai, China. Homozygous green fluorescent protein-expressing (GFP^+) mice were obtained from Model Animal Research Center of Nanjing University. *p27*$^{flox/flox}$ mice and *Nkx2.1-Cre* mice were purchased from the Jackson Laboratory (JAX #027328 and #008661), and bred and genotyped by PCR as recommended. Animals were maintained under a 12-h light/dark cycle with food and water provided *ad libitum*. Embryonic day 0.5 (E0.5) was defined as the date of sperm plug detection. Postnatal day 1 (P1) was defined as the date of birth. All experiments were conducted in accordance with Fudan University's guidelines for animal research and use.

Tissue dissection

Timed CD-1, *p27*-WT and *p27*-cKO embryos at E13.5 were used. Fetal brains were first cut coronally at a thickness of 250 μm with a vibratome (Leica VT1000S) in chilled oxygenated artificial cerebrospinal fluid. The medial ganglionic eminence (MGE) was dissected from slices (**Fig. 1O**) and transferred to DMEM-F12 (for culture; GIBCO, 11330032) or Leibovitz L-15 medium (for transplantation; GIBCO, 21083027) containing DNase I (100 μg/ml; Invitrogen, 18047019) and papain (20 μl/ml; Sigma, P3125). After incubation for 30 minutes at 37°C, dissociation was stopped by adding an equal volume of DMEM-F12 or L-15. Embryonic MGE explants were then mechanically dissociated into a single cell suspension. Dissociated cells were washed twice with 2 ml corresponding medium and pelleted by centrifugation (5 min, 300×g). Cells were re-suspended with medium for culture or L-15 and counted with a hemocytometer. For transplantation, cells were concentrated and re-suspended in L-15 medium to final density, and kept on ice until transplantation.

Cell culture

35 mm glass bottom culture dishes (MatTek, P35G-0-20-C) were coated with 0.05 mg/ml poly-D-lysine (Sigma-Aldrich, P7280), overnight at room temperature, air-dried, and then washed three times with ddH_2O. Dissociated MGE cells (1.2×10^4 cells per cm^2) were plated in the coated dishes in serum-free culture medium: DMEM/F-12, HEPES (Thermo Fisher, 11330032), 1% GlutaMAX (Thermo Fisher, 35050061), 2% B27 Supplement Minus Vitamin A (Thermo Fisher, 12587010), 1% N2 (Thermo Fisher, 17502048), 1% Penicillin-Streptomycin (Invitrogen, 15070-063), 10 ng/ml FGF2 (Invitrogen, PMG0031). Plated cells were incubated at 37°C in 5% CO_2 and 100% humidity.

Thymidine analogs

For in vivo studies, IdU (Sigma, 1336001), BrdU (Sigma, B5002) and EdU (Invitrogen, A1044) were dissolved at 5 mg/mL in sterile phosphate-buffered saline (PBS; pH 7.4). In E13.5 mice, thymidine analogs were administered by intraperitoneal injection at a dose of 50 mg/kg for IdU and BrdU, and 40 mg/kg for EdU, as described in **Fig. 1A**. For cell culture studies, IdU, BrdU and EdU were dissolved in anhydrous DMSO (Sigma, D5879) to make a 10 mM stock solution. IdU,

BrdU and EdU were subsequently added in culture medium for one hour (**Fig. 1F**). The final concentration of IdU, BrdU and EdU was 10 μM in culture medium.

Cell-cycle length calculation

Sequential injection of BrdU/EdU is a commonly used method for calculating cell-cycle length of progenitors in vivo(Ponti et al. 2013). In our experiments, pregnant mice received one injection of BrdU and one of EdU 2 h (T_i) later. Thirty minutes after EdU injection, we performed fixation and a tissue sample from each of these embryos was collected for genotyping. Brain sections from these embryos were used for immunostaining of BrdU, EdU and Ki67. We then counted the number of $BrdU^+/EdU^-$ cells and $Ki67^+$ cells in MGE. The average cell-cycle length of MGE was calculated by the formula: $Ti * \frac{Ki67+}{BrdU+EdU-} = Tc$.

Time-lapse video recording and cell lineage reconstruction

The time-lapse video recordings were captured by Perkin Elmer UltraView live cell imaging system. For long-term imaging, recording was started around 2 hours after plating. Clones were followed and imaged every 30 min over a period of 50 h. For the triple thymidine analog treatments, following recording, the cultured cells were fixed with 4% paraformaldehyde (PFA) in PBS for 2 h and then immunostained. The images were edited, analyzed, and selectively cropped into videos using Volocity Demo 5.5.1 software (Perkin Elmer), Imaris 7.4.2 (Bitplane) and Photoshop CS6 (Adobe). For lineage tree reconstructions, the time-lapse movies were replayed and the movement and divisions of each individual MGE cell were followed manually over time. To generate the lineage tree, the progeny of a single cell were tracked and their thymidine analog incorporation identified based on their specific morphologies and validated by the corresponding immunostaining data.

Transplantation

Concentrated cell suspensions (20–40 cells/nl) were injected into the cortical superficial layer of CD-1 mice at P1–P2 with a glass syringe (PCR micropipettes, Drummond Scientific, 5-000-1001-X10). There were four injection sites in the brain: posterior 1 mm from bregma, 1 mm lateral to the midline; anterior 1 mm from lambda, 2 mm lateral to the midline in each brain hemisphere. ~0.5 μl of cell solution was injected at each site. For *p27*-WT or *p27*-cKO MGE cell transplantation, pregnant mouse was administered BrdU by intraperitoneal injection at a dose of 50 mg/kg. Two hours later, we used one-to-one transplantation between the donor embryo and the host mouse. A tissue sample from each of these embryos was collected for genotyping after transplantation. Only host mice that received either *p27*-WT (*Nkx2.1-Cre*$^{-/-}$; *p27*$^{fl/fl}$) or *p27*-cKO (*Nkx2.1-Cre*$^{+/-}$; *p27*$^{fl/fl}$) MGE cells were analyzed.

Tissue collection and immunohistochemistry

Pregnant mice injected with thymidine analogs and transplanted mice at P30 were deeply anesthetized by 1% isoflurane and 0.5–1.0 ml/min oxygen. Embryos and transplanted mice were perfused intracardially with cold 4% PFA in PBS (pH 7.4). Embryonic brains and transplanted

adult brains were removed from the skulls, postfixed for 12 h at 4°C, washed in PBS and sectioned coronally at 30 μm on a vibratome (Leica VT1000S).

We first detected EdU in fixed culture cells and brain sections using Click-iT EdU Alexa 647 kit (Invitrogen, C10340) for 30 minutes at room temperature. The fixed culture cells and sections were then incubated with modified Click-iT EdU kit (Alexa Fluor 647 azide in this kit was replaced with 2 mM azidomethylphenylsulfide) for 30 minutes at room temperature (Liboska et al. 2012). After EdU detection and washing in PBS, the fixed culture cells and brain sections were incubated with 2N HCl for 1 h at room temperature, rinsed in 0.1M boric acid (pH 8.5) for 10 min and then in PBS. The cells and sections were first processed with PBS containing 10% donkey serum (Millipore, S30) and 0.5% Triton X-100 (Sigma, T8787) for 1.5 h at room temperature (pH 7.2), then incubated with the primary antibodies in PBS containing 5% donkey serum, 0.5% Triton X-100, and 0.05% sodium azide for 48 h at 4°C. After washing in PBST (0.1% Triton X-100 in PBS), the fixed culture cells and brain sections were incubated with secondary antibodies overnight at 4°C and subsequently washed in PBS.

The following primary antibodies were used: mouse anti-IdU (1:250, Sigma, SAB3701448), rat anti-BrdU (1:250, Abcam, ab6326), rabbit anti-Olig2 (1:100, Millipore, Ab9610), rabbit anti-cyclinD2 (1:300 Santa Cruz #sc-593), chicken anti-GFP (1:1000, Aves #1020), goat anti-somatostatin (1:250, Santa Cruz #sc-7819), rabbit anti-somatostatin (1:800, Abcam, ab64053), rabbit anti-parvalbumin (1:200, Abcam, ab11427), mouse anti-parvalbumin (1:1000, Millipore, MAB1572), mouse anti-pVimentin (1:1000, MBL, D076-3), mouse anti-γTubulin (1:1000, Sigma, T6557), rabbit anti-Ki67 (1:500, Abcam, ab15580), rabbit anti-PH3 (1:1000, Millipore, 06-570) and rabbit anti-cleaved caspase-3 (1:600, Cell signaling, 9661s). Secondary antibodies used were: donkey anti-mouse IgG Alexa Fluor 488 (1:250, Thermo Fisher, A-21202), donkey anti-rat IgG Alexa Fluor 568 (1:250, Abcam, ab175475), donkey anti-rabbit IgG Alexa Fluor 405 (1:250, Abcam, ab175651), donkey anti-goat IgG Alexa Fluor 405 (1:250, Abcam, ab175664), donkey anti-chicken IgY Alexa Fluor 488 (1:250, Jackson, #703-546-155), donkey anti-rabbit IgG Alexa Fluor 568 (1:250, Thermo Fisher, A10042), donkey anti-mouse IgG Alexa Fluor 647 (1:250, Thermo Fisher, A31571), donkey anti-rat IgG Alexa Fluor 488 (1:250, Thermo Fisher, A21208), and donkey anti-rabbit IgG Alexa Fluor 488 (1:250, Thermo Fisher, A21206).

Imaging and cell distribution analysis

Brain sections and cultured cells were scanned using Olympus FV3000 confocal microscope with a 20× or 60× objective. Digital images were balanced in brightness, contrast, and color with Imaris 7.4.2 (Bitplane). In the MGE, the VZ and SVZ boundary was confirmed by the distribution of EdU^+ cells. To precisely characterize the distribution of PSC and PLC in the MGE, we divided the MGE in coronal brain slices into six 30° sectors (**Fig. 1J**), sequentially labeled *A–F* from the dorsal to the ventral side. In a coronal section of embryonic brain, we defined the lowest points of the MGE on the eminence surface as points *a* and *b*, and the lowest points on the boundary between VZ and SVZ as points *a'* and *b'*. The SVZ/mantle boundary is delineated by the straight line *a'b'*. The center point (*o*) of a hemi-circle on line *a'b'* was determined, equidistant to *a* and *b*. Using the

hemi-circle, MGEs were divided into six 30° sectors.

In situ hybridization

E13.5 embryonic brains of *p27*-WT or *p27*-cKO mice were perfused intracardially with cold 4% PFA in PBS (pH 7.4), postfixed for 24 h at 4°C and dehydrated with 30% sucrose in Diethyl pyrocarbonate (DEPC)-treated PBS until the brains sank. Brains were coronally sectioned at a thickness of 10 μm. Probes (ACD, 499991) and RNA detection reagent kit (ACD, 322300) were bought from ACD company. Brain section pretreatment and p27 mRNA detection were performed according to the product specification.

3D reconstruction and dividing angle determination

Thirty micron coronal sections of E13.5 fetal brains were stained with mouse anti-pVimentin (1:1000; MBL D076-3), mouse anti-γTubulin (1:1000; Sigma T6557), and rabbit anti-PH3 (Millipore 06-570) (**Fig. 2E**). Z stacks with an interval of 0.5 μm were taken using Olympus FV3000 confocal microscope. After 3D reconstruction of the confocal stacks of a dividing cell with the Imaris software, four points were placed in the centers of neighboring cells at different surface positions around the targeted 3D-rendered cell, and two points were placed at the positions of the two centrosomes. The coordinates of the four points were used to determine the best-fitting plane by least squares method. The angle β between the vector connecting the two points marking the centrosomes and the normal vector of the plane was calculated, and 90° minus the angle β was used as the division angle α (**Fig. 2F**). All calculations were done using the python 3.x environment.

Data acquisition and analysis

Data are presented as mean ± SEM. Statistical analyses were performed using SigmaPlot 12.0 (Systat Software), GraphPad Prism 8 and SPSS 25 (IBM). The statistical difference between groups was determined by two-tailed unpaired *t* test, one-way ANOVA or two-way ANOVA with post-hoc Tukey HSD test. p values < 0.05 were considered to be statistically significant.

Supplemental Reference

Liboska R, Ligasová A, Strunin D, et al (2012) Most Anti-BrdU Antibodies React with 2′-Deoxy-5-Ethynyluridine — The Method for the Effective Suppression of This Cross-Reactivity. PLoS ONE 7:e51679. https://doi.org/10.1371/journal.pone.0051679

Ponti G, Obernier K, Guinto C, et al (2013) Cell cycle and lineage progression of neural progenitors in the ventricular-subventricular zones of adult mice. Proc Natl Acad Sci USA 110:E1045–E1054. https://doi.org/10.1073/pnas.1219563110

Supplemental Figure Legends

Figure S1. Distribution of $IdU^{+}BrdU^{-}EdU^{+}$ cells and $IdU^{+}BrdU^{-}EdU^{-}$ cells in thymidine analog-treated MGEs. (**A**–**D**) Representative images showing thymidine analog incorporation for $\Delta T = 4$ h , $\Delta T = 9$ h, $\Delta T = 12$ h, and $\Delta T = 14$ h at E13.5. Scale bar, 50 μm. (**E**–**H**) Distribution of $IdU^{+}BrdU^{-}EdU^{+}$ cells (red dots) and $IdU^{+}BrdU^{-}EdU^{-}$ cells (black dots) in A–D. (**I**–**L**) Representative images showing thymidine analog incorporation for $\Delta T = 4$ h, $\Delta T = 9$ h, $\Delta T = 12$ h, and $\Delta T = 14$ h at E15.5. Scale bar, 50 μm. (**M**–**P**) Distribution of $IdU^{+}BrdU^{-}EdU^{+}$ cells (red dots) and $IdU^{+}BrdU^{-}EdU^{-}$ cells (black dots) in I–L.

Figure S2. Cell-cycle length of cultured MGE progenitors exhibits remarkable heterogeneity and instability. (**A**) Schema showing the method of MGE dissection and culture. (**B**) Snapshots of time-lapse video showing clonal divisions. Cells were followed and imaged every 30 min. Time is given in hours:minutes. (**C**) The lineage tree of cell divisions reconstructed from the same clone shown in B. (**D**) Bar graph showing distribution of cell-cycle length based on time-lapse recording of cultured MGE progenitor cells at E13.5. (**E**) The cell-cycle length of cultured E13.5 and E15.5 MGE cells. (**F**) Four line charts representing four types of change in the cell-cycle length in three-round divisions: increase (red), decrease (blue), oscillation (green), and stable (grey). (**G**) The percentage of the four types of change in the cell-cycle length undergone by cultured MGE cells at E13.5. (**H**) Representative differential interference contrast (DIC), immunostained and merged images of thymidine analog-treated MGE cells after time-lapse recording. Scale bar, 100 μm. (**I**) Experimental outline in cultured MGE cells. (**J**) The cell-cycle length of $IdU^{+}BrdU^{-}EdU^{+}$ cells under different ΔT conditions. Each set of data come from three separate experiments. Data are presented as mean ± SEM.

Figure S3. The distribution of PSC and PLC in the MGE. (**A**) Quantification of the density of IdU^+BrdU^- cells in the VZ when ΔT = 4 h. (**B**) Quantification of the density of IdU^+BrdU^- cells in the VZ when ΔT = 12 h. (**C**) Quantification of the density of IdU^+BrdU^- cells in the SVZ when ΔT = 4 h. (**D**) Quantification of the density of IdU^+BrdU^- cells in the SVZ when ΔT = 12 h. (**E–H**) Representative images and distribution reconstructions of $IdU^+BrdU^-EdU^+$ cells and $IdU^+BrdU^-EdU^-$ cells in thymidine analog-treated MGE. Scale bar, 100 μm. (**I**) Schema depicting the lateral view of an E13.5 telencephalon. (**J** and **K**) The density of PSC and PLC in rostral MGE, central MGE and caudal MGE at E13.5. Data are presented as mean ± SEM. (**L**) Confocal image showing IdU, BrdU, EdU and Olig2 expression in thymidine analog-treated MGE (ΔT =12 h). Scale bar, 80 μm. (**M**) Histogram of the percentage of $IdU^+BrdU^-EdU^+Olig2^+$ cells in $IdU^+BrdU^-EdU^+$ cells among PSC and PLC located in the VZ. Data are presented as mean ± SEM. (**N**) Confocal image showing IdU, BrdU, EdU and CCND2 expression in thymidine analog-treated MGE (ΔT = 12 h). Scale bar, 80 μm. (**O**) Histogram of the percentage of $IdU^+BrdU^-EdU^+CCND2^+$ cells in $IdU^+BrdU^-EdU^+$ cells among PSC and PLC located in the SVZ. Data are presented as mean ± SEM. Photo Credit for Figure 2A and 2J: Yongchun Yu, Fudan University, China.

Figure S4. Thymidine analog-labeled MGE cells displayed normal interneuron morphology after transplantation. (**A** and **B**) Representative images showing thymidine analogs co-labeling with GFP. Cells were transplanted into the cortex of the host mouse at P1 and imaged at P30. Scale bar, 200 μm. (**C**) Magnified cell images from A and B. Cell 1 is $IdU^{-}BrdU^{-}EdU^{+}GFP^{+}$; cell 2 and 4 are $IdU^{+}BrdU^{+}EdU^{-}GFP^{+}$; cell 3 is $IdU^{+}BrdU^{-}EdU^{+}GFP^{+}$. (**D**) Morphology of cells 1–4 (red arrowheads).

Figure S5. ***p27*** **mRNA expression and effects on cortical interneuron apoptosis in p27-cKO mice.** (**A**) Magnified images are below, 1 and 1', 2 and 2', 3 and 3', 4 and 4' showing MGE, LGE, cortex and CGE, respectively. Scale bar, 250 μm. (**B** and **C**) Representative images of PV (green) immunostaining in the striatum from *p27*-WT and *p27*-cKO mice at P30. Scale bar, 200 μm. (**D**) Quantification of the density of PV^+ interneurons in the striatum of *p27*-WT and *p27*-cKO mice. Data are presented as mean ± SEM. (**E** and **F**) Representative images of SST (purple) immunostaining in the striatum from *p27*-WT and *p27*-cKO mice at P30. Scale bar, 200 μm. (**G**) Quantification of the density of SST^+ interneurons in the striatum of *p27*-WT and *p27*-cKO mice. Data are presented as mean ± SEM. (**H**) Representative image of cleaved caspase-3 immunostaining in the cortex. Scale bar, 50 μm. (**I**) Numbers of cleaved caspase-3^+ cells in the cortex of *p27*-WT and *p27*-cKO mice. Data are presented as mean ± SEM.

Figure S1

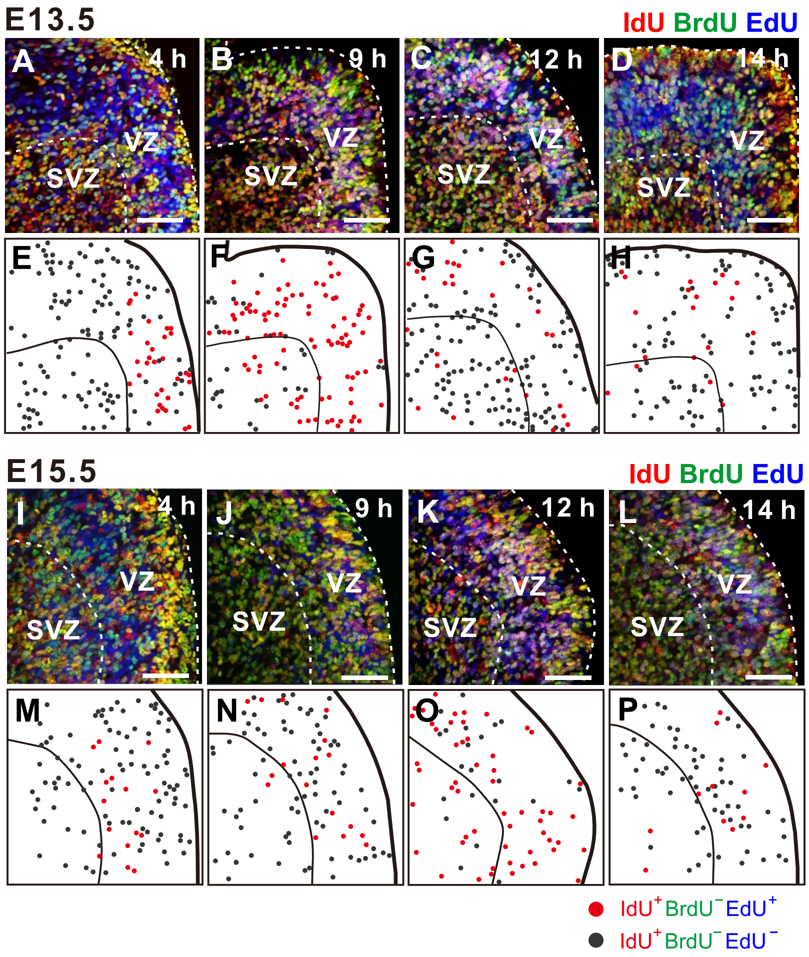
E13.5
IdU BrdU EdU
A
4 h
B
9 h
C
12 h
D
14 h
VZ
SVZ
E
F
G
H
E15.5
IdU BrdU EdU
I
4 h
J
9 h
K
12 h
L
14 h
M
N
O
P
IdU+ BrdU− EdU+
IdU+ BrdU− EdU−

Figure S2

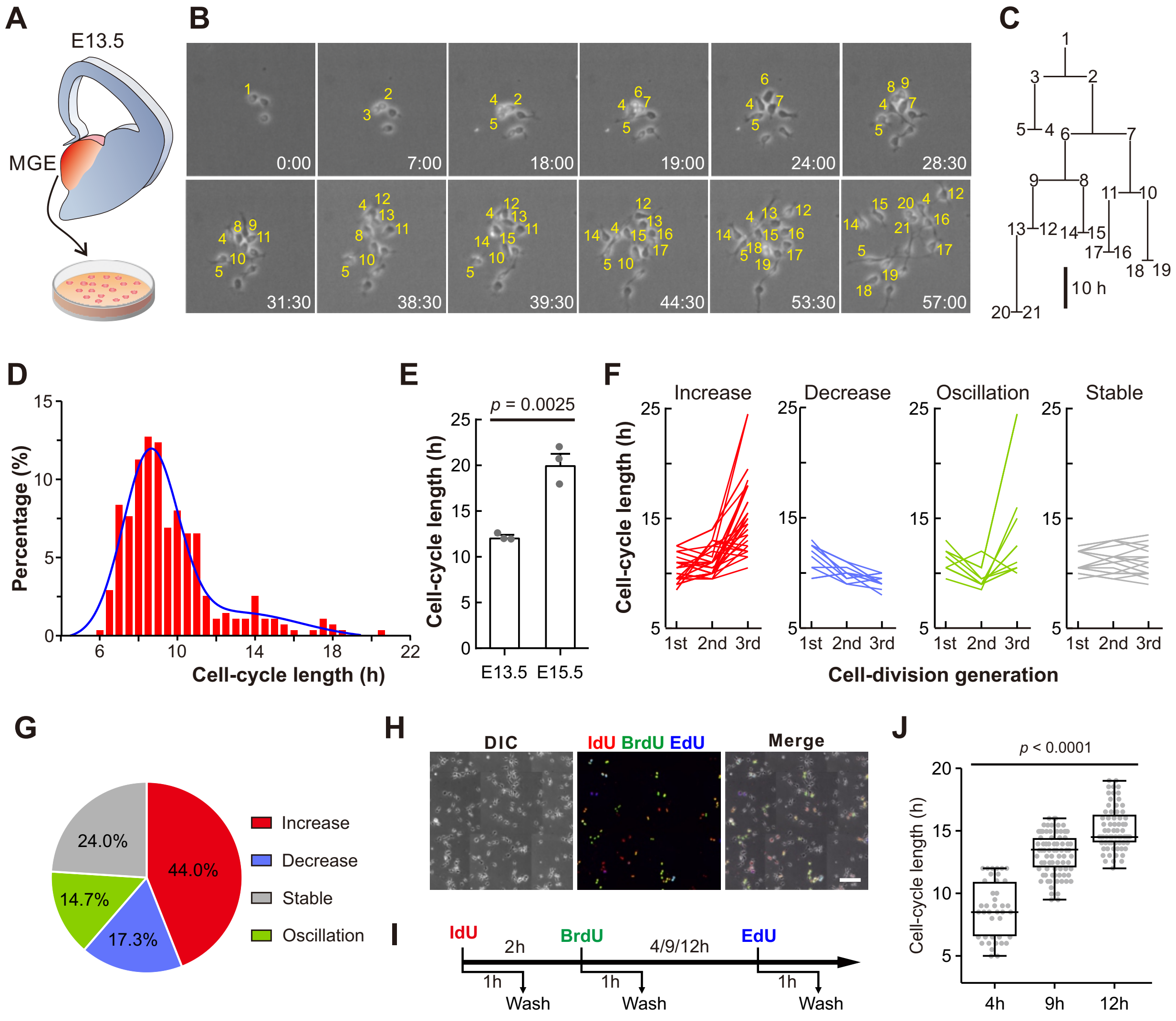

A
E13.5
MGE
B
0:00
7:00
18:00
19:00
24:00
28:30
31:30
38:30
39:30
44:30
53:30
57:00
C
10 h
D
Percentage (%)
Cell-cycle length (h)
E
p = 0.0025
Cell-cycle length (h)
E13.5
E15.5
F
Increase
Decrease
Oscillation
Stable
Cell-cycle length (h)
1st 2nd 3rd
Cell-division generation
G
Increase
Decrease
Stable
Oscillation
44.0%
17.3%
14.7%
24.0%
H
DIC
IdU BrdU EdU
Merge
I
IdU
2h
BrdU
4/9/12h
EdU
1h
Wash
J
p < 0.0001
Cell-cycle length (h)
4h
9h
12h

Figure S3

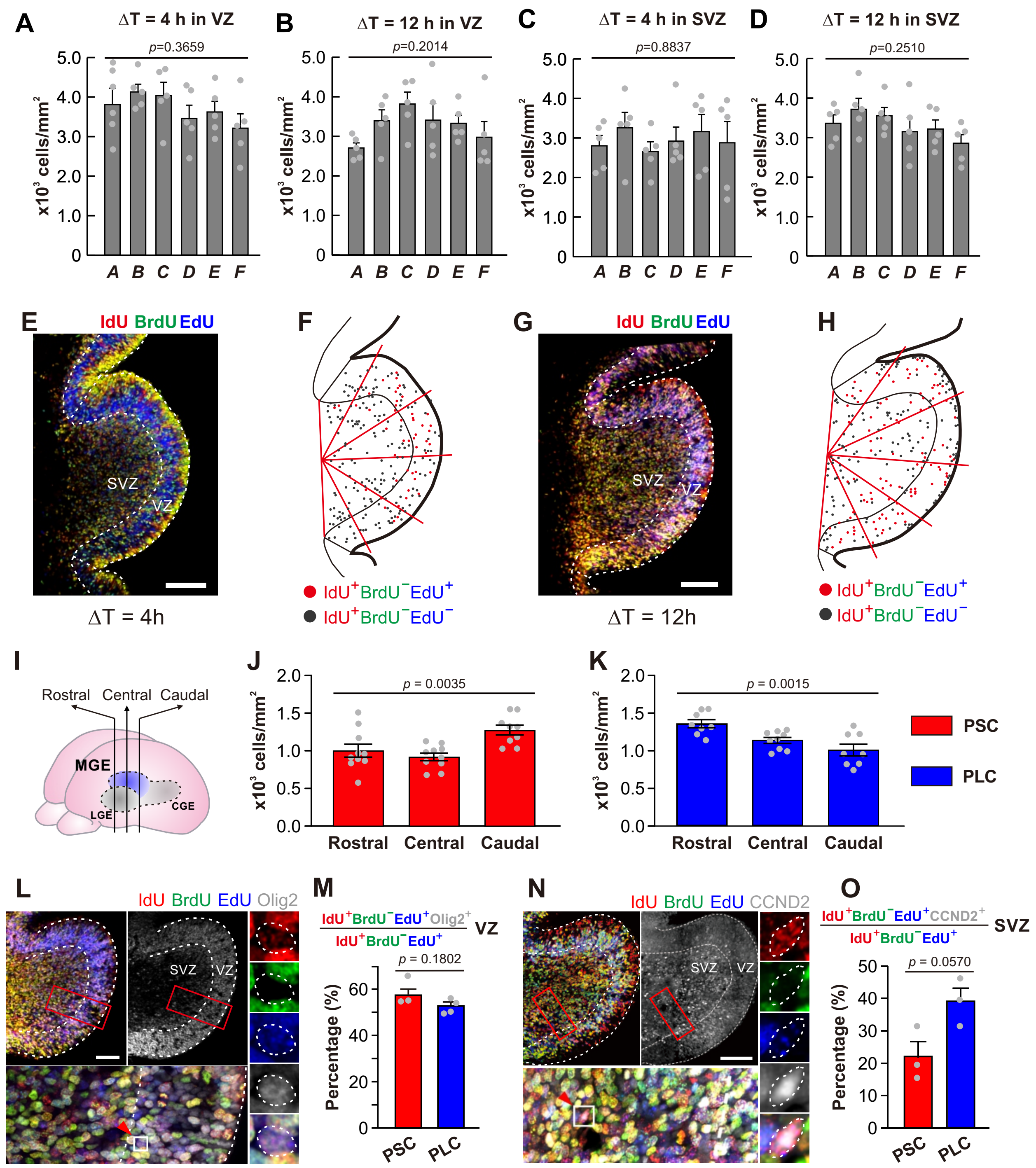

A
ΔT = 4 h in VZ
p=0.3659
B
ΔT = 12 h in VZ
p=0.2014
C
ΔT = 4 h in SVZ
p=0.8837
D
ΔT = 12 h in SVZ
p=0.2510
x10³ cells/mm²
A B C D E F
E
IdU BrdU EdU
SVZ
VZ
ΔT = 4h
F
IdU⁺BrdU⁻EdU⁺
IdU⁺BrdU⁻EdU⁻
G
IdU BrdU EdU
SVZ
VZ
ΔT = 12h
H
IdU⁺BrdU⁻EdU⁺
IdU⁺BrdU⁻EdU⁻
I
Rostral Central Caudal
MGE
LGE
CGE
J
p = 0.0035
K
p = 0.0015
Rostral Central Caudal
PSC
PLC
L
IdU BrdU EdU Olig2
SVZ
VZ
M
IdU⁺BrdU⁻EdU⁺Olig2⁺ / IdU⁺BrdU⁻EdU⁺ VZ
p = 0.1802
Percentage (%)
PSC PLC
N
IdU BrdU EdU CCND2
SVZ
VZ
O
IdU⁺BrdU⁻EdU⁺CCND2⁺ / IdU⁺BrdU⁻EdU⁺ SVZ
p = 0.0570
Percentage (%)
PSC PLC

Figure S4

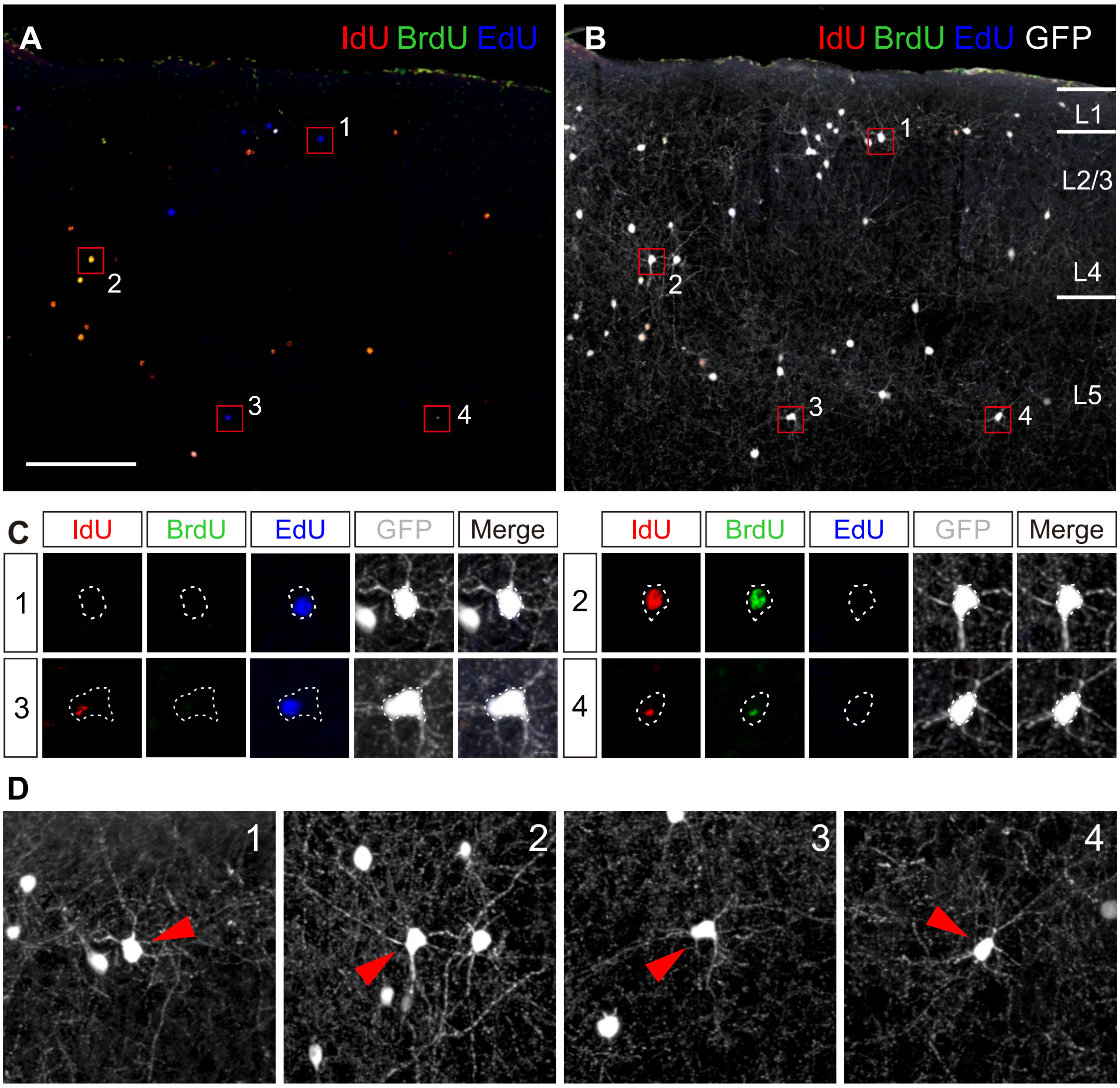
A
IdU BrdU EdU
1
2
3
4
B
IdU BrdU EdU GFP
L1
L2/3
L4
L5
C
IdU
BrdU
EdU
GFP
Merge
1
2
3
4
D
1
2
3
4

Figure S5

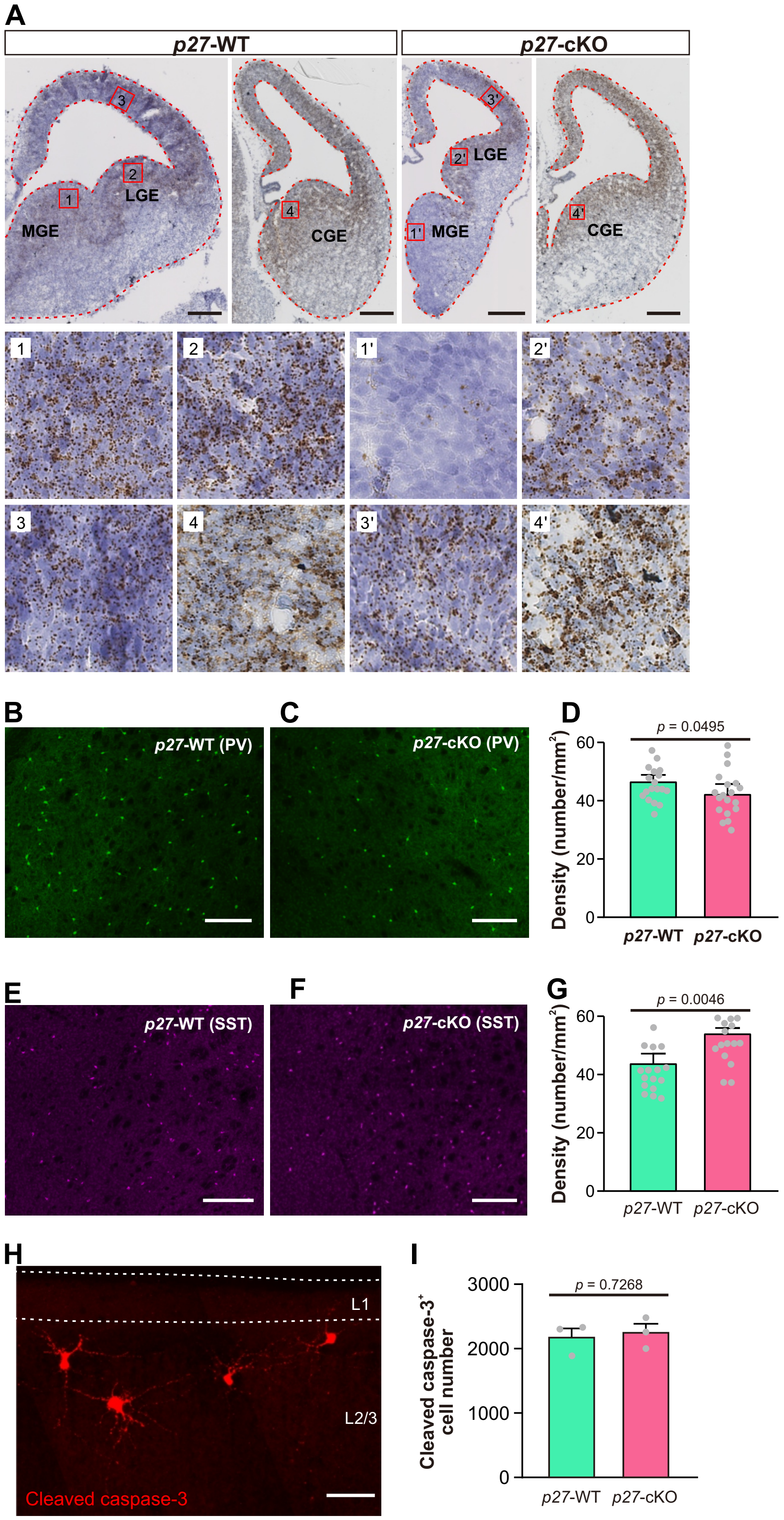
A
p27-WT
p27-cKO
MGE
LGE
CGE
1
2
3
4
1'
2'
3'
4'
B
C
D
p27-WT (PV)
p27-cKO (PV)
p = 0.0495
Density (number/mm²)
60
40
20
0
p27-WT
p27-cKO
E
F
G
p27-WT (SST)
p27-cKO (SST)
p = 0.0046
H
L1
L2/3
Cleaved caspase-3
I
p = 0.7268
Cleaved caspase-3⁺ cell number
3000
2000
1000
0

Supplemental Tables

Table S1. Detailed statistical analysis, data, exact sample numbers and *p* values in Fig. 1 and Fig. S1–Fig. S4.

Figure number	Test used	*n* number	Descriptive Stats (mean ± SEM)	*p* value
Fig. 1D	one-way ANOVA with *post-hoc* Tukey HSD test	n = 6 mice	4 h: 16.65 ± 1.45%	$F_{(3,19)}$ = 76.0900, $p < 0.0001$; Note that although the *p* value is significant across the 4 groups, we performed *post-hoc* Tukey HSD test and significant difference was found between: 4 h and 9 h ($p = 0.0010$); 4 h and 12 h ($p = 0.0403$); 9 h and 12 h ($p = 0.0010$); 9 h and 14 h ($p = 0.0010$); 12 h and 14 h ($p = 0.0105$).
		n = 3 mice	9 h: 61.95 ± 4.83%	
		n = 8 mice	12 h: 24.11 ± 1.68%	
		n = 6 mice	14 h: 15.03 ± 1.45%	
Fig. 1E	one-way ANOVA with *post-hoc* Tukey HSD test	n = 5 mice	4 h: 8.61 ± 1.10%	$F_{(3,16)}$ = 101.3000, $p < 0.0001$; Note that although the *p* value is significant across the 4 groups, we performed *post-hoc* Tukey HSD test and significant difference was found between: 4 h and 9 h ($p < 0.0001$); 4 h and 12 h ($p < 0.0001$); 9 h and 12 h ($p < 0.0001$); 9 h and 14 h ($p = 0.7533$); 12 h and 14 h ($p < 0.0001$).
		n =6 mice	9 h: 32.35 ± 2.66%	
		n = 4 mice	12 h: 63.85 ± 1.80%	
		n = 5 mice	14 h: 33.47 ± 2.02%	
**Figure number**	**ΔT**	***n* number**		**Descriptive Stats (mean ± SEM)**
Fig. 1I	4 h	n = 61 divisions from three separate experiments		Once: 100 ± 0%<br>Twice: 0 ± 0%

	12 h	n = 97 divisions from three separate experiments	Once: 97.13 ± 2.87%<br>Twice: 2.87 ± 2.87%
	14 h	n = 97 divisions from three separate experiments	Once: 65.95 ± 5.66%<br>Twice: 34.05 ± 5.66%

**Figure number**	**Test used**	***n* number**	**Descriptive Stats (mean ± SEM, hours)**	***p* value**
Fig. S2E	two-tailed unpaired *t* test	n = 6 mice	E13.5: 12.20 ± 0.20	$p = 0.0025$
		n = 6 mice	E15.5: 20.11 ± 1.16	
Fig. S2J	two-tailed unpaired *t* test	n=40 cells	4h: 8.69 ± 2.27	$p < 0.0001$
		n=81 cells	9h: 13.17 ± 1.67	
		n=68 cells	12h: 15.15 ± 1.74	

**Figure number**	**Test used**	***n* number**	**Descriptive Stats (mean ± SEM, cells/mm^2)**	***p* value**
Fig. S3A	one-way ANOVA	n = 5 mice	*A* sector:<br>3809.3 ± 414.3<br>*B* sector:<br>4128.8 ± 196.2<br>*C* sector:<br>4035.1 ± 337.6<br>*D* sector:<br>3459.3 ± 335.2<br>*E* sector:<br>3622.3 ± 269.7<br>*F* sector:<br>3212.3 ± 362.1	$F_{(5,29)} = 1.1416$, $p = 0.3659$

Fig. S3B	one-way ANOVA	n = 5 mice	*A* sector:<br>2706.7 ± 128.4<br>*B* sector:<br>3392.7 ± 277.1<br>*C* sector:<br>3816.6 ± 302.4<br>*D* sector:<br>3408.4 ± 419.3<br>*E* sector:<br>3329.5 ± 203.9<br>*F* sector:<br>2976.8 ± 396.1	$F_{(5,29)} = 1.5881$,<br>$p = 0.2014$
Fig. S3C	one-way ANOVA	n = 5 mice	*A* sector:<br>2801.1 ± 262.9<br>*B* sector:<br>3256.9 ± 389.6<br>*C* sector:<br>2658.5 ± 243.9<br>*D* sector:<br>2919.7 ± 355.6<br>*E* sector:<br>3158.7 ± 436.6<br>*F* sector:<br>2877.5 ± 537.3	$F_{(5,29)} = 0.3398$,<br>$p = 0.8837$
Fig. S3D	one-way ANOVA	n = 5 mice	*A* sector:<br>3360.8 ± 213.1<br>*B* sector:<br>3715.6 ± 278.4<br>*C* sector:<br>3552.2 ± 209.5<br>*D* sector:<br>3149.5 ± 355.0<br>*E* sector:<br>3215.4 ± 230.1<br>*F* sector:<br>2855.2 ± 218.0	$F_{(5,29)} = 1.4255$,<br>$p = 0.2510$
**Figure number**	**Test used**	***n* number**	**Descriptive Stats (mean ± SEM)**	***p* value**

Fig. 1K	one-way ANOVA with *post-hoc* Tukey HSD test	n = 5 mice	Density (cells/mm^2)<br>*A* sector: 575.7 ± 24.3<br>*B* sector: 1579.4 ± 148.4<br>*C* sector: 1973.1 ± 256.0<br>*D* sector: 1210.4 ± 106.9<br>*E* sector: 944.4 ± 100.4<br>*F* sector: 202.9 ± 50.4	$F_{(5,29)} = 22.5303$,<br>$p < 0.0001$;<br>Note that although the *p* value is significant across the 6 regions, we performed *post-hoc* Tukey HSD test and significant difference was found between:<br>*A* and *B* ($p = 0.0010$);<br>*A* and *C* ($p = 0.0010$);<br>*A* and *D* ($p = 0.0330$);<br>*B* and *E* ($p = 0.0330$);<br>*B* and F ($p = 0.0010$);<br>*C* and *D* ($p = 0.0071$);<br>*C* and *E* ($p = 0.0010$);<br>*C* and *F* ($p = 0.0010$);<br>*D* and *F* ($p = 0.0010$);<br>*E* and *F* ($p = 0.0092$).
Fig. 1L	one-way ANOVA with *post-hoc* Tukey HSD test	n = 5 mice	Density (cells/mm^2)<br>*A* sector: 1321.8 ± 227.8<br>*B* sector: 602.6 ± 120.9<br>*C* sector: 512.6 ± 64.3<br>*D* sector: 683.6 ± 72.2<br>*E* sector: 1343.0 ± 177.4<br>*F* sector: 1410.7 ± 212.2	$F_{(5,29)} = 6.9549$,<br>$p = 0.0004$;<br>Note that although the *p* value is significant across the 6 regions, we performed *post-hoc* Tukey HSD test and significant difference was found between:<br>*A* and *B* ($p = 0.0403$);<br>*A* and *C* ($p = 0.0163$);<br>*B* and *E* ($p = 0.0330$);<br>*B* and *F* ($p = 0.0165$);<br>*C* and *E* ($p = 0.0131$);<br>*C* and *F* ($p = 0.0064$);<br>*D* and *F* ($p = 0.0373$).
Fig. 1M	one-way ANOVA with *post-hoc* Tukey HSD test	n = 5 mice	Density (cells/mm^2)<br>*A* sector: 191.1 ± 71.7<br>*B* sector: 364.6 ± 63.2<br>*C* sector: 413.3 ± 91.2<br>*D* sector: 253.3 ± 48.5<br>*E* sector: 242.8 ± 67.9<br>*F* sector: 44.5 ± 31.1	$F_{(5,29)} = 4.0431$,<br>$p = 0.0084$;<br>Note that although the *p* value is significant across the 6 regions, we performed *post-hoc* Tukey HSD test

				and significant difference was found between:<br>*B* and *F* ($p = 0.0211$);<br>*C* and *F* ($p = 0.0061$).
Fig. 1N	one-way ANOVA with *post-hoc* Tukey HSD test	n = 5 mice	Density (cells/mm2)<br>*A* sector: 585.0 ± 119.9<br>*B* sector: 461.5 ± 60.9<br>*C* sector: 528.2 ± 85.4<br>*D* sector: 679.8 ± 64.1<br>*E* sector: 1072.3 ± 67.0<br>*F* sector: 987.1 ± 140.2	$F_{(5,29)} = 7.1423$,<br>$p = 0.0003$;<br>Note that although the *p* value is significant across the 6 regions, we performed *post-hoc* Tukey HSD test and significant difference was found between:<br>*A* and *E* ($p = 0.0144$);<br>*B* and *E* ($p = 0.0015$);<br>*B* and *F* ($p = 0.0073$);<br>*C* and *E* ($p = 0.0052$);<br>*C* and *F* ($p = 0.0234$).
Fig. S3J	one-way ANOVA with *post-hoc* Tukey HSD test	n = 3 mice	Density (cells/mm2)<br>Rostral: 1000 ± 90.0<br>Central: 920 ± 50.0<br>Caudal: 1280 ± 70	$F_{(2,26)} = 7.0810$,<br>$p = 0.0035$;<br>Note that although the *p* value is significant across the 6 regions, we performed *post-hoc* Tukey HSD test and significant difference was found between:<br>*Rostral* and *Central* ($p = 0.4172$);<br>*Rostral* and *Caudal* ($p = 0.0231$);<br>*Central* and *Caudal* ($p = 0.0004$);
Fig. S3K	one-way ANOVA with *post-hoc* Tukey HSD test	n = 3 mice	Density (cells/mm^2)<br>Rostral: 1350 ± 50<br>Central: 1130 ± 40<br>Caudal: 1000 ± 80	$F_{(2,22)} = 8.9130$,<br>$p = 0.0015$;<br>Note that although the *p* value is significant across the 6 regions, we performed *post-hoc* Tukey HSD test and significant

				difference was found between:<br>*Rostral* and *Central* (p = 0.0038);<br>*Rostral* and *Caudal* (p = 0.0025);<br>*Central* and *Caudal* (p = 0.1680);
Fig. S3M	two-tailed unpaired *t* test	n = 3 mice	PSC: 57.48 ± 2.61%	p = 0.1802
		n = 4 mice	PLC: 52.82 ± 1.73%	
Fig. S3O	two-tailed unpaired *t* test	n = 3 mice	PSC: 21.84 ± 4.83%	p = 0.0570
		n = 3 mice	PLC: 38.89 ± 4.26%	

**Figure number**	**Test used**	***n* number**	**Descriptive Stats (mean ± SEM)**	***p* value**
Fig. 1Q	two-tailed unpaired *t* test	n = 3 mice	PSC: 22.87 ± 2.88%	p = 0.0043
		n = 4 mice	PLC: 51.64 ± 4.77%	
Fig. 1R	two-tailed unpaired *t* test	n = 3 mice	PSC: 54.84 ± 2.01%	p = 0.0009
		n = 4 mice	PLC: 31.26 ± 2.42%	
Fig. 1S	two-tailed unpaired *t* test	n = 3 mice	PSC: 30.72 ± 1.42%	p <0.0001
		n = 3 mice	PLC: 64.36 ± 0.80%	
Fig. 1T	two-tailed unpaired *t* test	n = 3 mice	PSC: 52.16% ± 4.48%	p =0.0087
		n = 3 mice	PLC: 26.10% ± 3.09%	

Table S2. Detailed statistical analysis, data, exact sample numbers and *p* values in Fig. 2 and Fig. S5.

**Figure number**	**Test used**	***n* number**	**Descriptive Stats (mean ± SEM)**	***p* value**
Fig. 2B	two-tailed unpaired *t* test	n = 3 mice	*p27*-WT: 15.34 ± 0.34	*p* = 0.0009
		n = 3 mice	*p27*-cKO: 11.22 ± 0.32	
Fig. 2C	two-tailed unpaired *t* test	n = 3 mice	*p27*-WT: 16.65 ± 1.45%	*p* < 0.0001
		n = 3 mice	*p27*-cKO: 32.71 ± 1.26%	
Fig. 2D	two-tailed unpaired *t* test	n = 3 mice	*p27*-WT: 24.11 ± 1.69%	*p* = 0.0108
		n = 3 mice	*p27*-cKO: 17.38 ± 1.57%	
Fig. 2H	two-tailed unpaired *t* test	n = 4 mice	*p27*-WT: 40.82 ± 1.54	*p* = 0.0137
		n = 4 mice	*p27*-cKO: 35.5 ± 1.50	
**Figure number**	**Test used**	***n* number**	**Descriptive Stats (mean ± SEM)**	***p* value**
Fig. 2J	two-way ANOVA (mice type X layer) with *post-hoc* Tukey HSD test	n=3 mice	L2/3 *p*27-WT:<br>219.5 ± 6.63<br>L2/3 *p*27-cKO:<br>189.8 ± 8.27<br>L4 *p*27-WT:<br>268.5 ± 13.05<br>L4 *p*27-cKO:<br>218.7 ± 20.78<br>L5 *p*27-WT:<br>274.1 ± 16.42<br>L5 *p*27-cKO:<br>231.2 ± 10.19<br>L6 *p*27-WT:<br>137.7 ± 7.61<br>L6 *p*27-cKO:<br>109.8 ± 7.19	Mice type:<br>F (1, 18) = 8.330,<br>*p* = 0.0098<br>Layer:<br>F (2.557, 46.02) = 79.50,<br>*p* < 0.0001<br>Interaction between mice type and layer:<br>F (3, 54) = 0.6427,<br>*p* = 0.5909<br><br>*Post-hoc* Tukey HSD test showed significant differences only at layer 6 *p27*-WT vs. *p27*-cKO:<br>*p* = 0.0161;

Fig. 2K	two-way ANOVA (mice type X layer) with *post-hoc* Tukey HSD test	n=3 mice	L2/3 *p*27-WT: 91.38 ± 6.67 L2/3 *p*27-cKO: 116.2 ± 3.41<br>L4 *p*27-WT: 80.67 ± 11.29<br>L4 *p*27-cKO: 118.4 ± 9.82<br>L5 *p*27-WT: 181.2 ± 27.01<br>L5 *p*27-cKO: 308.4 ± 36.41<br>L6 *p*27-WT: 85.22 ± 9.99<br>L6 *p*27-cKO: 159.7 ± 22.78	Mice type:<br>F (1, 16) = 10.75,<br>*p* = 0.0047<br>Layer:<br>F (1.589, 25.42) = 42.79,<br>*p* < 0.0001<br>Interaction between mice type and layer:<br>F (3, 48) = 4.747,<br>*p* = 0.0056<br><br>*Post-hoc* Tukey HSD test showed significant differences at each of layer:<br>L2/3 *p27*-WT vs. *p27*-cKO:<br>*p* = 0.0043;<br>L4 *p27*-WT vs. *p27*-cKO:<br>*p* = 0.0225;<br>L5 *p27*-WT vs. *p27*-cKO:<br>*p* = 0.0127;<br>L6 *p27*-WT vs. *p27*-cKO:<br>*p* = 0.0086;
Fig. 2L	two-way ANOVA (mice type X layer) with *post-hoc* Tukey HSD test	n=3 mice	L2/3 *p*27-WT: 225.9 ± 16.40 L2/3 *p*27-cKO: 207.3 ± 12.24<br>L4 *p*27-WT: 359.6 ± 20.47<br>L4 *p*27-cKO: 272.1 ± 12.75<br>L5 *p*27-WT: 292.0 ± 16.13<br>L5 *p*27-cKO: 241.4 ± 15.16<br>L6 *p*27-WT: 144.6 ± 2.43<br>L6 *p*27-cKO: 128.2 ± 7.36	Mice type:<br>F (1, 24) = 8.963,<br>*p* = 0.0063<br>Layer:<br>F (2.826, 67.82) = 98.61,<br>*p* < 0.0001<br>Interaction between mice type and layer:<br>F (3, 72) = 4.691,<br>*p*=0.0048<br><br>*Post-hoc* Tukey HSD test showed significant differences only at layer 4 *p27*-WT vs. *p27*-cKO:<br>*p* = 0.0013;

Fig. 2M	two-way ANOVA (mice type X layer) with *post-hoc* Tukey HSD test	n=3 mice	L2/3 *p*27-WT: 135.0 ± 7.61 L2/3 *p*27-cKO: 166.3 ± 10.05<br>L4 *p*27-WT: 245.9 ± 20.67<br>L4 *p*27-cKO: 305.2 ± 17.10<br>L5 *p*27-WT: 205.8 ± 21.93<br>L5 *p*27-cKO: 325.3 ± 16.31<br>L6 *p*27-WT: 86.22 ± 6.66<br>L6 *p*27-cKO: 121.0 ± 4.28	Mice type:<br>F (1, 24) = 13.42,<br>*p* = 0.0012<br>Layer:<br>F (2.048, 49.16) = 152.4,<br>*p* < 0.0001<br>Interaction between mice type and layer:<br>F (3, 72) = 8.770,<br>*p* < 0.0001<br><br>*Post-hoc* Tukey HSD test showed significant differences at each of layer:<br>L2/3 *p27*-WT vs. *p27*-cKO: *p* = 0.0203;<br>L4 *p27*-WT vs. *p27*-cKO: *p* = 0.0369;<br>L5 *p27*-WT vs. *p27*-cKO: *p* = 0.0002;<br>L6 *p27*-WT vs. *p27*-cKO: *p* = 0.0002;
Fig. 2N	two-way ANOVA (mice type X layer) with *post-hoc* Tukey HSD test	n=3 mice	L2/3 *p*27-WT: 178.1 ± 16.37 L2/3 *p*27-cKO: 194.2 ± 5.87<br>L5 *p*27-WT: 219.2 ± 13.08<br>L5 *p*27-cKO: 225.7 ± 10.01<br>L6 *p*27-WT: 134.1 ± 9.63<br>L6 *p*27-cKO: 133.0 ± 22.08	Mice type:<br>F (1, 12) = 0.2242,<br>*p* = 0.6444<br>Layer:<br>F (1.476, 17.71) = 34.61,<br>*p* < 0.0001<br>Interaction between mice type and layer:<br>F (2, 24) = 0.3243,<br>*p* = 0.7261

Fig. 2O	two-way ANOVA (mice type X layer) with *post-hoc* Tukey HSD test	n=3 mice	L2/3 *p*27-WT: 121.4 ± 9.59 L2/3 *p*27-cKO: 118.1 ± 4.10<br>L5 *p*27-WT: 217.8 ± 19.44<br>L5 *p*27-cKO: 234.8 ± 21.05<br>L6 *p*27-WT: 131.2 ± 18.58<br>L6 *p*27-cKO: 163.7 ± 35.94	Mice type:<br>F (1, 12) = 0.5468,<br>*p* = 0.4739<br>Layer:<br>F (1.557, 18.68) = 19.32,<br>*p* < 0.0001<br>Interaction between mice type and layer:<br>F (2, 24) = 0.5094,<br>*p* = 0.6072
**Figure number**	**Test used**	***n* number**	**Descriptive Stats (mean ± SEM)**	***p* value**
Fig. S5D	two-tailed unpaired *t* test	n = 3 mice	*p27*-WT: 46.56 ± 1.67	*p* = 0.0495
		n = 3 mice	*p27*-cKO: 41.60 ± 1.78	
Fig. S5G	two-tailed unpaired *t* test	n = 3 mice	*p27*-WT: 43.77 ± 2.81	*p* = 0.0046
		n = 3 mice	*p27*-cKO: 54.44 ± 2.12	
Fig. S5I	two-tailed unpaired *t* test	n = 3 mice	*p27*-WT: 2172 ± 246.6	*p* = 0.7268
		n = 3 mice	*p27*-cKO: 2247 ± 241.2	
**Figure number**	**Test used**	***n* number**	**Descriptive Stats (mean ± SEM)**	***p* value**
Fig. 2S	two-tailed unpaired *t* test	n = 3 mice	*p27*-WT: 83.43 ± 2.33%	*p* = 0.7244
		n = 3 mice	*p27*-cKO: 81.23 ± 5.35%	
Fig. 2T	two-tailed unpaired *t* test	n = 3 mice	*p27*-WT: 50.82 ± 4.37%	*p* = 0.0080
		n = 3 mice	*p27*-cKO: 26.80 ± 2.19%	
Fig. 2U	two-tailed unpaired *t* test	n = 3 mice	*p27*-WT: 32.61 ± 3.04%	*p* = 0.0100
		n = 3 mice	*p27*-cKO: 54.43 ± 4.20%	
